# Electronic Properties of Carbon Nanobelts Predicted by Thermally-Assisted-Occupation DFT

**DOI:** 10.3390/nano11092224

**Published:** 2021-08-29

**Authors:** Sonai Seenithurai, Jeng-Da Chai

**Affiliations:** 1 Department of Physics, National Taiwan University, Taipei 10617, Taiwan; seenithurai@gmail.com; 2 Center for Theoretical Physics and Center for Quantum Science and Engineering, National Taiwan University, Taipei 10617, Taiwan; 3 Physics Division, National Center for Theoretical Sciences, Taipei 10617, Taiwan

**Keywords:** TAO-DFT, carbon nanobelts, multi-reference character, electronic properties

## Abstract

Accurate prediction of properties of large-scale multi-reference (MR) electronic systems remains difficult for traditional computational methods (e.g., the Hartree–Fock theory and Kohn–Sham density functional theory (DFT)). Recently, thermally-assisted-occupation (TAO)-DFT has been demonstrated to offer reliable description of electronic properties of various large-scale MR electronic systems. Consequently, in this work, TAO-DFT is used to unlock the electronic properties associated with C-Belt[*n*] (i.e., the carbon nanobelts containing *n* fused 12-membered carbon rings). Our calculations show that for all the system sizes reported (*n* = 4–24), C-Belt[*n*] have singlet ground states. In general, the larger the size of C-Belt[*n*], the more pronounced the MR character of ground-state C-Belt[*n*], as evident from the symmetrized von Neumann entropy and the occupation numbers of active TAO-orbitals. Furthermore, the active TAO-orbitals are delocalized along the circumference of C-Belt[*n*], as evident from the visualization of active TAO-orbitals.

## 1. Introduction

Carbon is one of the richest elements in forming a number of allotropes. Carbon forms 0D, 1D, and 2D nanostructures, such as the well-known C${}_{60}$ fullerenes (buckyballs), carbon nanotubes (CNTs), and graphene [1,2]. It is also possible to form a plethora of hybrid carbon nanostructures by combining these well-known nanostructures with other nanostructures. The diverse nature originates from the sp-, $sp^{2}$-, and $sp^{3}$-hybridized carbon atoms as well as the combinations of different types of hybridization. Owing to the unique and diverse properties, carbon-based nanomaterials have been applied in many fields, including nanotechnology, electronics, optics, biotechnology, and materials science. In addition to these applications, carbon nanostructures have also drawn attention to their beauty, creating playgrounds for testing different phenomena. Moreover, in carbon-based molecular science, polycyclic aromatic hydrocarbons (PAHs), for example, can have astronomical and biological importance as well.

Among a variety of carbon nanostructures, although the C${}_{60}$ fullerenes, CNTs, and graphene have been well studied, belt-shaped carbon nanomaterials have been relatively under-reported [3,4,5,6,7,8,9,10,11]. In fact, belt-shaped carbon nanomaterials can be promising because of their important role in supramolecular chemistry. The structure of belt-shaped carbon molecules may offer new possibilities for the synthesis of chemical compounds with various functional groups. Moreover, owing to the deformation of typical sp- or $sp^{2}$-type orbitals, belt-shaped carbon molecules (i.e., unconventional π-electron systems) can have fascinating properties for some applications (e.g., optoelectronic applications).

Over the past few years, there has been a renewed interest in synthesizing and applying belt-shaped carbon molecules, where the belt structure is formed with fused benzene rings [4,5,11]. Belt-shaped carbon molecules include [*n*]cycloparaphenylene ([*n*]CPP) in which the benzene rings are connected by covalent bonds [4,5], belt[*n*]arene (also called *n*-cyclacene [7]) where the benzene rings are fused in a linear fashion, a carbon nanobelt containing the benzene rings fused in a non-linear fashion, etc. These molecules can act as unique macrocyclic hosts, and can also be used in molecular recognition and self-assembly. For example, belt-shaped carbon molecules (e.g., [12]CPP, which has already been synthesized [4]) can be considered as the building blocks of CNTs.

Armchair hydrocarbon belts (e.g., [*n*]CPP), zigzag hydrocarbon belts (e.g., belt[*n*]arene or *n*-cyclacene), zigzag carbon nanobelts, etc. are the common names used for carbon nanobelts [11]. The majority of carbon nanobelts consist of only benzene rings (i.e., 6-membered carbon rings). As is well known, carbon forms various nanostructures with pentagon (5-membered ring) and hexagon (6-membered ring) motifs. However, various evidences have shown that carbon nanostructures with motifs other than 5- and 6-membered rings can also be found. For example, 4- and 8-membered CNTs [12], buckyballs with motifs other than 6-membered rings [13], etc. have been proposed. In addition, belt[*n*]arene[*n*]tropilidenes, consisting of 6- and 7-membered rings, have been realized [14]. Accordingly, it seems possible to design and synthesize the carbon nanobelts with motifs other than 5- and 6-membered rings.

Very recently, there have been many attempts to synthesize and apply carbon nanobelts [4,5,11]. Several belt-shaped carbon molecules, which are entirely made of fused 6-membered carbon rings, have been recently synthesized. These carbon nanobelts can be used as templates or seeds to synthesize structurally well-defined CNTs [15]. Most of the carbon nanobelts are made of fused benzene rings or the benzene rings connected by covalent bonds. The bonding of rings may be linear or sideways, offering numerous possibilities for generating carbon nanobelts with different structures and properties [11,14], and hence unlocking new applications.

**Figure 1:**
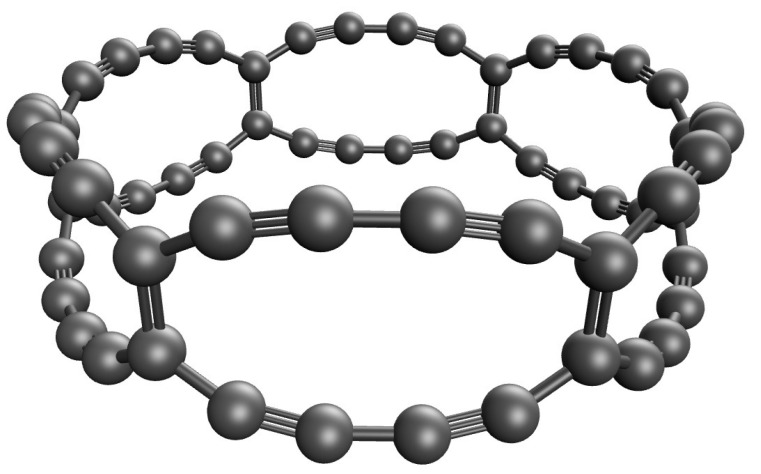
Geometry of C-Belt[6], containing 6 fused 12-membered carbon rings forming a closed loop.

Among various carbon nanobelts, in this work, we focus on the carbon nanobelts consisting of *n* fused 12-membered carbon rings (as inspired by the work of Estrada and Simón-Manso [6]), denoted as C-Belt[*n*], as illustrated in Figure 1. These carbon nanobelts need some attention to understand their properties and potential applications. As of now, it has been difficult to synthesize C-Belt[*n*], probably because of the high strain and multi-reference (MR) character (as will be shown and discussed later) associated with these carbon nanobelts. According to the recent KS-DFT (Kohn–Sham density functional theory) [16] calculations on C-Belt[*n*], these carbon nanobelts (also called Escherynes) should be highly stable [6]. However, several important electronic properties (e.g., singlet-triplet gap, MR character, etc.) of C-Belt[*n*] remain unavailable. Moreover, the main challenge in the reliable prediction of electronic properties of belt-shaped molecules should be due to the MR character associated with these molecules. Based on our previous studies on belt-shaped and closely related molecules, such as cyclacenes [7], cyclic boron nanoribbons [17], and cyclic carbon chains [18], we speculate that C-Belt[*n*] could also possess MR character. It is well-known that KS-DFT employing the commonly used exchange-correlation energy functionals can lead to enormous errors in the predicted properties of MR electronic systems, due to the unsuccessful incorporation of static correlation into these exchange-correlation energy functionals [19,20]. Consequently, the properties of C-Belt[*n*] should be explored by more reliable computational methods, such as MR computational methods [21,22,23,24,25,26,27,28,29,30]. However, conventional MR computational methods are intractable for large electronic systems (e.g., C-Belt[*n*] with large values of *n*), limiting the applications of these MR methods to relatively small electronic systems.

Aiming to unlock the ground-state properties of large-scale MR electronic systems, TAO-DFT (thermally-assisted-occupation density functional theory) [31] has been formulated in recent years. TAO-DFT can offer approximate static correlation in a cost-effective way, i.e., via an entropy contribution consisting of the TAO (thermally-assisted-occupation) orbital occupation numbers produced by the Fermi–Dirac distribution with a fictitious temperature θ, even when adopting the unsophisticated LDA (local density approximation) exchange-correlation and θ-dependent energy functionals [31], wherein the energy density depends only on the local electron density. More complicated semilocal and hybrid functionals in TAO-DFT have been proposed as well [32,33,34]. Moreover, the simple system-independent θ scheme [31,32,33] and the more sophisticated system-dependent θ scheme [35] in TAO-DFT have also been devised for general applications. Recently, TAO-DFT has been combined with AIMD (*ab initio* molecular dynamics) to further unlock the dynamical properties of large-scale MR electronic systems [36]. Because of these recent developments, TAO-DFT has been successfully used to study several challenging radical molecules and nanomaterials, giving rise to the electronic properties [7,8,9,10,17,18,37,38,39,40,41], properties related to hydrogen storage [42,43,44], and vibrational frequencies [36,45].

To avoid any possible confusion with finite-temperature DFT (FT-DFT) (first proposed by Mermin [46] and powered by the Mermin–Kohn–Sham Equation [16]), we briefly describe the difference between TAO-DFT and FT-DFT. As emphasized in Ref. [31], TAO-DFT and FT-DFT may look similar, but they differ greatly in physical meanings. TAO-DFT is an electronic structure method for studying the ground-state properties of an electronic system at zero electronic temperature, while FT-DFT is developed for the thermodynamic properties of an electronic system in thermal equilibrium at a real electronic temperature $\theta_{\mathrm{real}}$. Furthermore, in TAO-DFT, the real electronic temperature is absolute zero (i.e., $\theta_{\mathrm{real}}$ = 0), and the fictitious temperature θ, which is related to the strength of static correlation associated with the electronic system at absolute zero, can be nonvanishing, while in FT-DFT, the real electronic temperature and the fictitious temperature are the same (i.e., they are both equal to $\theta_{\mathrm{real}}$). In other words, for the ground-state properties of electronic systems at zero electronic temperature (i.e., $\theta_{\mathrm{real}}$ = 0), FT-DFT is the same as KS-DFT, while TAO-DFT (with a nonvanishing fictitious temperature θ) can differ from KS-DFT. Note also that TAO-DFT (with a vanishing fictitious temperature, i.e., θ = 0) reduces to KS-DFT.

As a consequence, we use TAO-DFT to compute the electronic properties of C-Belt[*n*] (*n* = 4–24), reporting the singlet–triplet gaps, fundamental gaps and the associated vertical ionization potentials and electron affinities, symmetrized von Neumann entropy, occupation numbers of active TAO-orbitals, and visualization of active TAO-orbitals in this study.

## 2. Computational Details

Numerical results are obtained with TAO-LDA [31], which is TAO-DFT with the LDA exchange-correlation and θ-dependent energy functionals, where the recommended fictitious temperature θ = 7 mhartree [31] is used. All calculations are carried out with Q-Chem 4.4 [47], using the 6-31G(d) basis set.

## 3. Results and Discussion

### 3.1. Singlet-Triplet Gap

The nature of the ground state of a molecule (e.g., whether the molecule has a singlet or triplet ground state) can be understood from the singlet-triplet (ST) gap. Knowledge of the ST gap is also essential for many chemical processes [48,49]. Several research efforts have been made to get this quantity accurately. Experimentally, the ST gap can be obtained from phosphorescence measurements under cryogenic conditions [50,51,52]. However, for a large molecule with pronounced MR character, it remains difficult to measure the ST gap properly. Reliable theoretical calculations on the ST gap can be necessary to fill the gap. The calculation of ST gap is also demanding in understanding the singlet fission phenomenon, which can guide and motivate experimentalists.

First, we calculate the ST gap ($E_{\mathrm{ST}}=E_{T}-E_{S}$) of C-Belt[*n*] as the difference between the lowest triplet energy $E_{T}$ and the lowest singlet energy $E_{S}$ of C-Belt[*n*], using spin-unrestricted TAO-LDA, where the $E_{T}$ and $E_{S}$ are evaluated on the respective optimized geometries.

**Figure 2:**
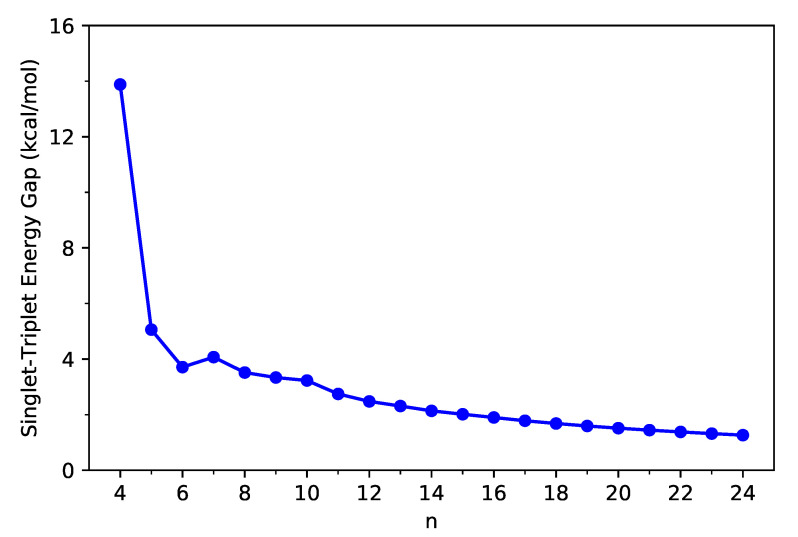
Singlet–triplet gap of C-Belt[*n*], computed using spin-unrestricted TAO-LDA.

As shown in Figure 2, the ground states of all the C-Belt[*n*] reported are singlet states (refer to Table S1 in Supplementary Information (SI) as well). The ST gap decreases drastically from *n* = 4 to *n* = 5, and for n≥7, the ST gap decreases, in a monotonic manner, with the system size *n*. Since MR electronic systems are often characterized by small ST gaps, the larger C-Belt[*n*] (e.g., n≥5), which have very small ST gaps (e.g., less than 6 kcal/mol), are likely to possess MR character.

Here, we assess a well-known exact symmetry requirement related to MR electronic systems. For the singlet state of a MR electronic system, unphysical symmetry-breaking solutions obtained from spin-unrestricted KS-DFT with the commonly used exchange-correlation energy functionals are frequently found [19,20,31,53]. In fact, the spin-restricted and spin-unrestricted calculations using an exact theory should yield the same energy for the singlet state of the MR electronic system. In order to check whether spin-unrestricted TAO-LDA yields any unphysical symmetry-breaking solutions, we also calculate the spin-restricted TAO-LDA energies, which are evaluated on the corresponding optimized geometries, for the lowest singlet states of C-Belt[*n*]. The aforementioned exact symmetry requirement is found to be indeed satisfied by TAO-LDA (i.e., the spin-restricted and spin-unrestricted energies, computed using TAO-LDA, are the same within the numerical precision considered) for all the C-Belt[*n*] reported.

### 3.2. Fundamental Gap and the Associated Vertical Ionization Potential and Electron Affinity

Ionization potential (also called ionization energy) and electron affinity play an important role in several chemical processes. For a neutral molecule, the vertical ionization potential refers to the change in energy when an electron is removed from the molecule (without altering the geometry of neutral molecule), the electron affinity refers to the change in energy when an electron is added to the molecule (without altering the geometry of neutral molecule), and their difference leads to the fundamental gap.

Following these definitions, with multiple energy-difference spin-unrestricted TAO-LDA calculations on the ground-state geometry of C-Belt[*n*], we obtain the vertical ionization potential (${\mathrm{IP}}_{v}=E_{N-1}-E_{N}$), the vertical electron affinity (${\mathrm{EA}}_{v}=E_{N}-E_{N+1}$), and the fundamental gap ($E_{g}={\mathrm{IP}}_{v}-{\mathrm{EA}}_{v}$) of C-Belt[*n*], where $E_{N-1}$, $E_{N}$, and $E_{N+1}$ are the energies of the cationic, neutral, and anionic states, respectively, of C-Belt[*n*].

**Figure 3:**
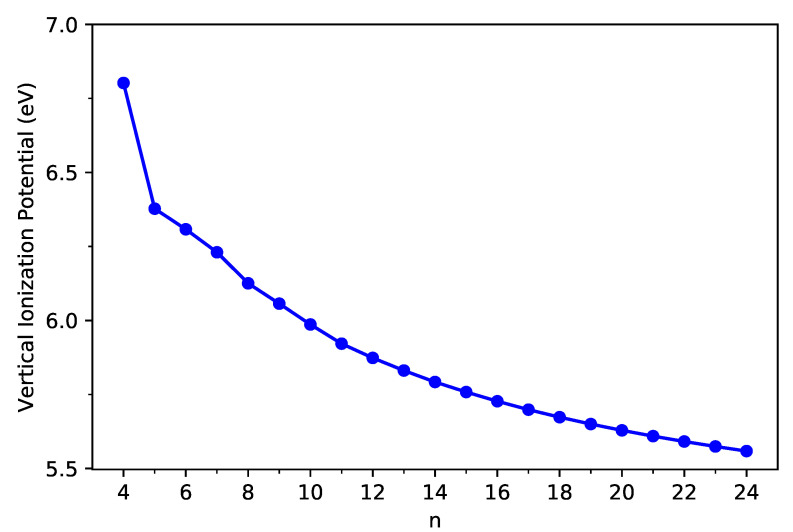
Vertical ionization potential of ground-state C-Belt[*n*], computed using spin-unrestricted TAO-LDA.

**Figure 4:**
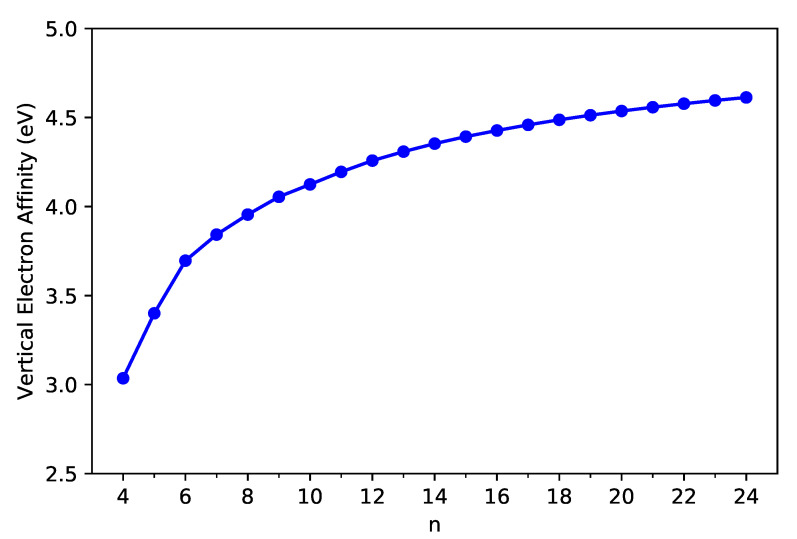
Vertical electron affinity of ground-state C-Belt[*n*], computed using spin-unrestricted TAO-LDA.

**Figure 5:**
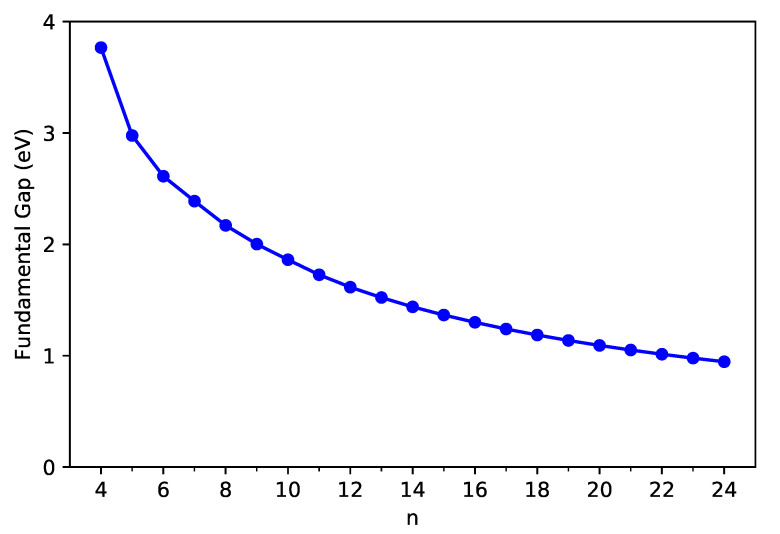
Fundamental gap of ground-state C-Belt[*n*], computed using spin-unrestricted TAO-LDA.

As shown in Figure 3 and Figure 4, the ${\mathrm{IP}}_{v}$ decreases monotonically, and the ${\mathrm{EA}}_{v}$ increases monotonically with *n* for all the C-Belt[*n*] reported. According to the definitions above, the fact that the monotonic decrease in ${\mathrm{IP}}_{v}$ and the monotonic increase in ${\mathrm{EA}}_{v}$ with the size of C-Belt[*n*] implies that the $E_{g}$ (see Figure 5) should decrease in a monotonic manner with *n* (refer to Table S2 in SI as well). The fundamental gaps of C-Belt[*n*] with *n* = 5–22 cover the range of ideal energy gaps (1–3 eV) for optoelectronic devices, and hence these carbon nanobelts can be considered as potential candidates for nanophotonics applications.

### 3.3. Symmetrized Von Neumann Entropy

The symmetrized von Neumann entropy [53] (see, e.g., Equation (6) of Ref. [18]),

(1) $$S_{\mathrm{vN}}=-\frac{1}{2}\sum_{\sigma =\alpha ,\beta }\sum_{i=1}^{\infty }\left\{f_{i,\sigma }\;\ln\left(f_{i,\sigma }\right)+\left(1-f_{i,\sigma }\right)\;\ln\left(1-f_{i,\sigma }\right)\right\},$$

which naturally emerges in the formulation of TAO-DFT (cf., the entropy contribution in TAO-DFT) [31,32,33], is an important quantity to understand the strength of MR character associated with an electronic system. In Equation (1), the occupation number $f_{i,\sigma }$ (ranging from 0 to 1) of the $i^{\mathrm{th}}$σ-spin (e.g., up-spin or down-spin) TAO-orbital, computed using spin-unrestricted TAO-DFT [31,32,33], approximately yields the occupation number of the $i^{\mathrm{th}}$σ-spin natural orbital [54]. According to its definition, the symmetrized von Neumann entropy is vanishingly small for an electronic system with non-radical character, and can be very large for an electronic system with pronounced MR character.

**Figure 6:**
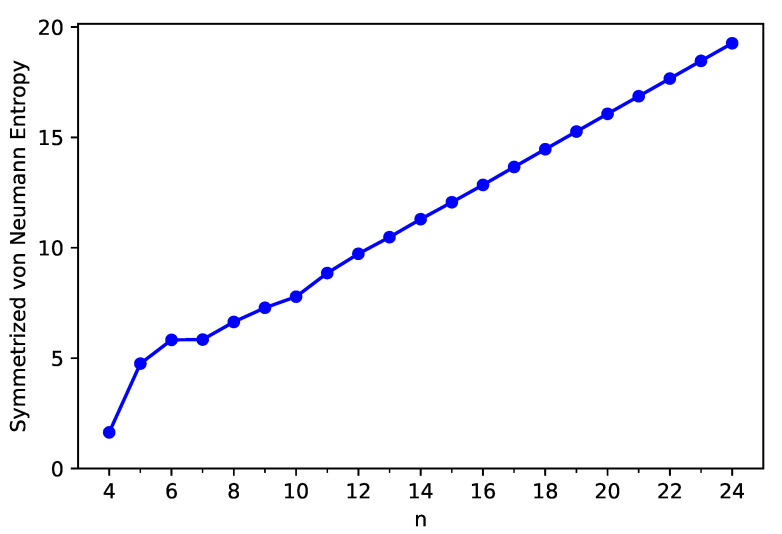
Symmetrized von Neumann entropy of ground-state C-Belt[*n*], computed using spin-unrestricted TAO-LDA.

To assess the strength of MR character associated with these carbon nanobelts, spin-unrestricted TAO-LDA calculations are performed to get the symmetrized von Neumann entropy $S_{\mathrm{vN}}$ of ground-state C-Belt[*n*]. As the size of C-Belt[*n*] increases, the $S_{\mathrm{vN}}$ (see Figure 6) increases in a monotonic manner (refer to Table S2 in SI as well). Moreover, the $S_{\mathrm{vN}}$ value is small for *n* = 4, suggesting that C-Belt[4] can be a non-radical molecule. By contrast, the $S_{\mathrm{vN}}$ values are rather large (e.g., larger than 4) for *n* = 5–24, suggesting that the larger C-Belt[*n*] (e.g., n≥5) can exhibit MR character whose strength generally increases with *n*.

### 3.4. Occupation Numbers of Active TAO-Orbitals

To understand the origin of the increase of $S_{\mathrm{vN}}$ with increasing size of C-Belt[*n*], we plot the occupation numbers of active TAO-orbitals, such as the HOMO−9, HOMO−8, …, HOMO, LUMO, …, LUMO+8, and LUMO+9, of ground-state C-Belt[*n*], computed using spin-restricted TAO-LDA. For the ground state (i.e., the lowest singlet state) of C-Belt[*n*] (with *N* electrons), the ${\left(N/2\right)}^{\mathrm{th}}$ TAO-orbital is taken as the HOMO, and the ${\left(N/2+1\right)}^{\mathrm{th}}$ TAO-orbital is taken as the LUMO, and so forth. For brevity, in this work, we denote HOMO as H, LUMO as L, and so on. Note that the TAO-orbital occupation numbers (TOONs) in TAO-DFT [31,32,33] can be regarded as the approximate natural orbital occupation numbers (NOONs) [54].

**Figure 7:**
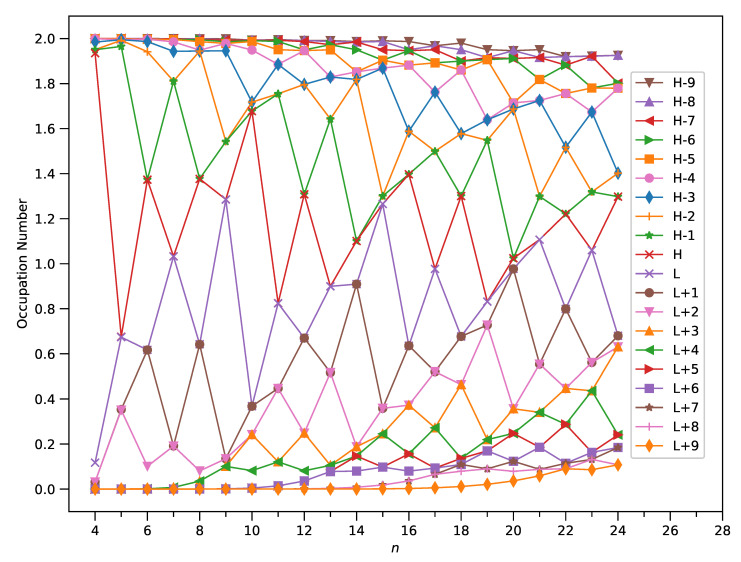
Occupation numbers of active TAO-orbitals (H − 9, H − 8, …, H − 1, H, L, L+1, …, L + 8, and L + 9) of ground-state C-Belt[*n*], computed using spin-restricted TAO-LDA.

As presented in Figure 7, the TOONs of ground-state C-Belt[*n*] clearly reveal the MR character associated with these carbon nanobelts. For example, C-Belt[4], with all the TOONs being either close to 0 or 2, is a non-radical molecule, showing consistency with the small $S_{\mathrm{vN}}$ value. Furthermore, C-Belt[7], with two TOONs being close to 1 and the remaining TOONs being either close to 0 or 2, exhibits diradical nature. In general, as *n* increases, C-Belt[*n*] possesses more TOONs deviating greatly from 0 and 2, giving rise to a more pronounced MR character and hence a larger value of $S_{\mathrm{vN}}$.

### 3.5. Visualization of Active TAO-Orbitals

**Figure 8:**
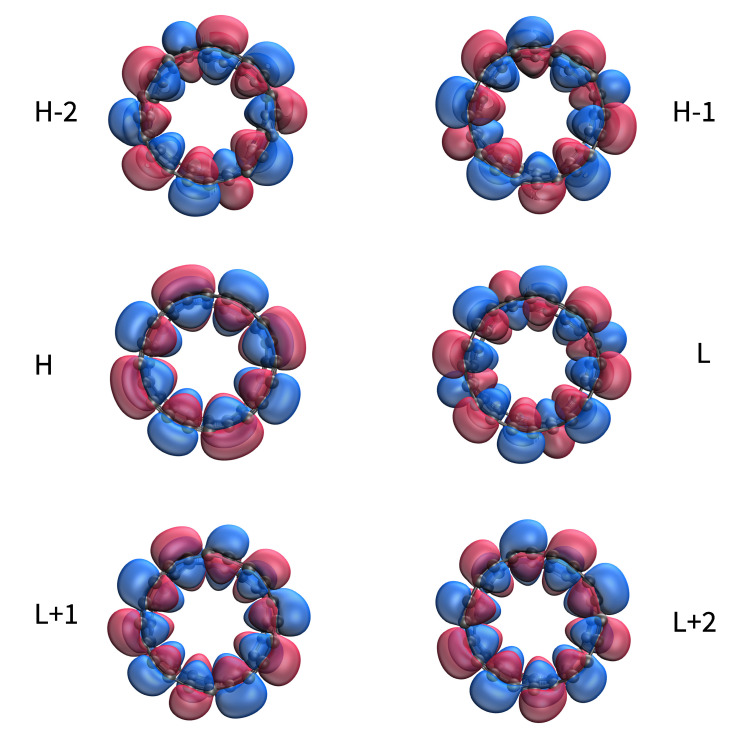
Visualization of active TAO-orbitals, such as H−2 (1.951), H−1 (1.950), H (1.935), L (0.117), L + 1 (0.032), and L + 2 (0.032), of ground-state C-Belt[4] at an isovalue of 0.02 e/Å${}^{3}$, computed using spin-restricted TAO-LDA. Numbers in parentheses show the TOONs.

In Figure 8, we present the visualization of active TAO-orbitals (H−2, H−1, H, L, L + 1, and L + 2) of ground-state C-Belt[4], computed using spin-restricted TAO-LDA (also refer to Figures S1–S3 in SI for the visualization of active TAO-orbitals of ground-state C-Belt[*n*] (*n* = 8, 12, and 16)). As shown, the active TAO-orbitals are delocalized over the circumference of C-Belt[*n*], showing similar features as have been observed in other belt-shaped molecules, such as the cyclacenes [7], cyclic boron nanoribbons [17], and cyclic carbon chains [18]. The presence of delocalized active TAO-orbitals over the C-Belt[*n*] implies that these carbon nanobelts can have high electrical conductivity [55,56].

## 4. Conclusions

In conclusion, we have presented a detailed computational study on the electronic properties of C-Belt[*n*] for *n* = 4–24. By using TAO-DFT [31], we have obtained the ST gaps, fundamental gaps and the associated vertical ionization potentials and electron affinities, symmetrized von Neumann entropy, occupation numbers of active TAO-orbitals, and visualization of active TAO-orbitals of C-Belt[*n*].

According to the symmetrized von Neumann entropy and the occupation numbers of active TAO-orbitals, in general, the larger C-Belt[*n*] should have more pronounced MR character, playing an important role in determining their electronic properties. Because of the MR character associated with these carbon nanobelts, KS-DFT with commonly used exchange-correlation energy functionals would be insufficient to predict the properties of the larger C-Belt[*n*] (e.g., n≥5) in a reliable manner. Due to the computational complexity, conventional MR computational methods would be intractable, given the large size of C-Belt[*n*]. Relative to conventional KS-DFT and MR computational methods, TAO-DFT is especially crucial for the study of large-scale MR electronic systems, such as the series of C-Belt[*n*] studied here.

Because of the MR character, it can be difficult to synthesize C-Belt[*n*] using conventional synthesis methods. However, owing to the recent growth in the synthesis methods of belt-shaped molecules, it can be anticipated that C-Belt[*n*] could be realized in the near future. As a follow-up to this work, it would be interesting to further study the energetics and thermal stability of C-Belt[*n*] using TAO-DFT-based AIMD simulations [36], and assess these properties for potential applications. Moreover, in this work, we have reported only the electronic properties of C-Belt[*n*], computed using the commonly used TAO-LDA (with θ = 7 mhartree) [31]. Certainly, it will be interesting to further examine how these electronic properties and other properties (e.g., structural properties) vary with different θ values (e.g., θ = 0 for KS-DFT, other system-independent θ values [31,32,33] as well as system-dependent θ values [35]) and density functionals (e.g., semilocal [32] and hybrid [33] functionals) in TAO-DFT. It is worth mentioning that other efficient electronic structure methods for strong static correlation [57,58] may also be employed here. In addition to C-Belt[*n*] (i.e., the carbon nanobelts consisting of *n* fused 12-membered carbon rings) reported in this work, the electronic properties of closely related carbon allotropes, such as the carbon nanobelts consisting of *n* fused 8-, 10-, and 14-membered carbon rings, can also be promising for certain applications. We plan to pursue some of these directions in the near future.

## Supplementary Materials

The following are available online at https://www.mdpi.com/article/10 .3390/nano11092224/s1, Figure S1: Visualization of active TAO-orbitals, such as H−2 (1.945), H−1 (1.377), H (1.377), L (0.642), L+1 (0.641), and L+2 (0.081), of ground-state C-Belt[8] at an isovalue of 0.02 e/Å${}^{3}$, computed using spin-restricted TAO-LDA. Numbers in parentheses show the TOONs, Figure S2: Visualization of active TAO-orbitals, such as H−2 (1.796), H−1 (1.308), H (1.308), L (0.670), L+1 (0.670), and L+2 (0.249), of ground-state C-Belt[12] at an isovalue of 0.02 e/Å${}^{3}$, computed using spin-restricted TAO-LDA. Numbers in parentheses show the TOONs, Figure S3: Visualization of active TAO-orbitals, such as H−2 (1.588), H−1 (1.397), H (1.397), L (0.636), L+1 (0.636), and L+2 (0.373), of ground-state C-Belt[16] at an isovalue of 0.02 e/Å${}^{3}$, computed using spin-restricted TAO-LDA. Numbers in parentheses show the TOONs, Table S1: Singlet-triplet gap $E_{\mathrm{ST}}$ (in kcal/mol) of C-Belt[*n*], computed using spin-unrestricted TAO-LDA, Table S2: Vertical ionization potential ${\mathrm{IP}}_{v}$ (in eV), vertical electron affinity ${\mathrm{EA}}_{v}$ (in eV), fundamental gap $E_{g}$ (in eV), and symmetrized von Neumann entropy $S_{\mathrm{vN}}$ of ground-state C-Belt[*n*], computed using spin-unrestricted TAO-LDA.
