# Supplementary material for: Electronic Properties of Carbon Nanobelts Predicted by Thermally-Assisted-Occupation DFT
Source: Nanomaterials (Basel). 2021 Aug 29;11(9):2224. doi: 10.3390/nano11092224 (PMC8465987; doi:10.3390/nano11092224)
Supplement: Supplementary file 1 [file nanomaterials-11-02224-s001.zip › nanomaterials-1346485-supplementary.pdf]

Supplementary Information to: Electronic Properties of Carbon Nanobelts Predicted by Thermally-Assisted-Occupation DFT

Sonai Seenithurai[1] and Jeng-Da Chai[1,2,3,*]

[1] *Department of Physics, National Taiwan University, Taipei 10617, Taiwan*

[2] *Center for Theoretical Physics and Center for Quantum Science and Engineering, National Taiwan University, Taipei 10617, Taiwan*

[3] *Physics Division, National Center for Theoretical Sciences, Taipei 10617, Taiwan*

* Author to whom correspondence should be addressed. Electronic mail: jdchai@phys.ntu.edu.tw

FIGURES

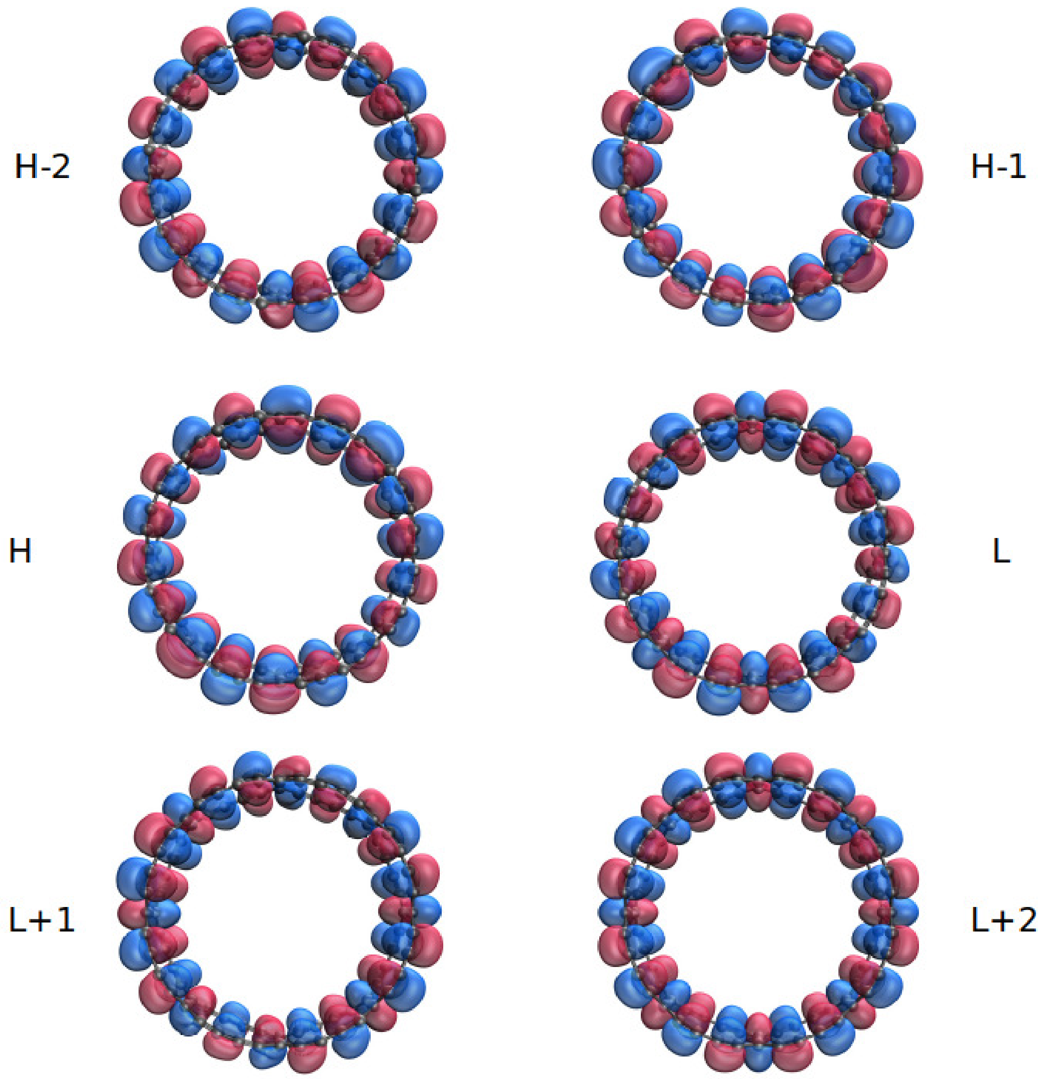

FIG. S1. Visualization of active TAO-orbitals, such as H−2 (1.945), H−1 (1.377), H (1.377), L (0.642), L+1 (0.641), and L+2 (0.081), of ground-state C-Belt[8] at an isovalue of 0.02 e/Å^3, computed using spin-restricted TAO-LDA. Numbers in parentheses show the TOONs.

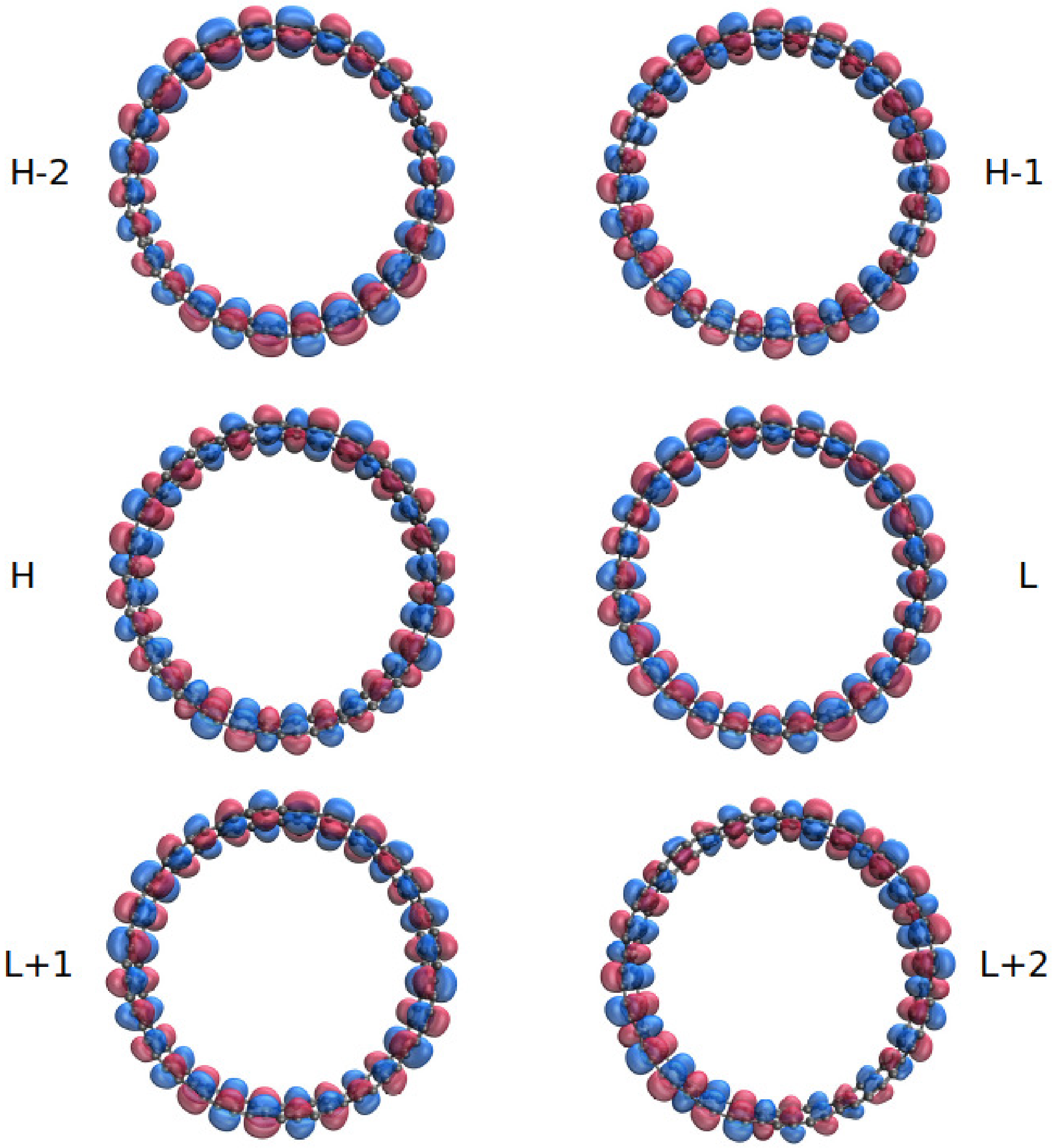

FIG. S2. Visualization of active TAO-orbitals, such as H−2 (1.796), H−1 (1.308), H (1.308), L (0.670), L+1 (0.670), and L+2 (0.249), of ground-state C-Belt[12] at an isovalue of 0.02 e/Å^3, computed using spin-restricted TAO-LDA. Numbers in parentheses show the TOONs.

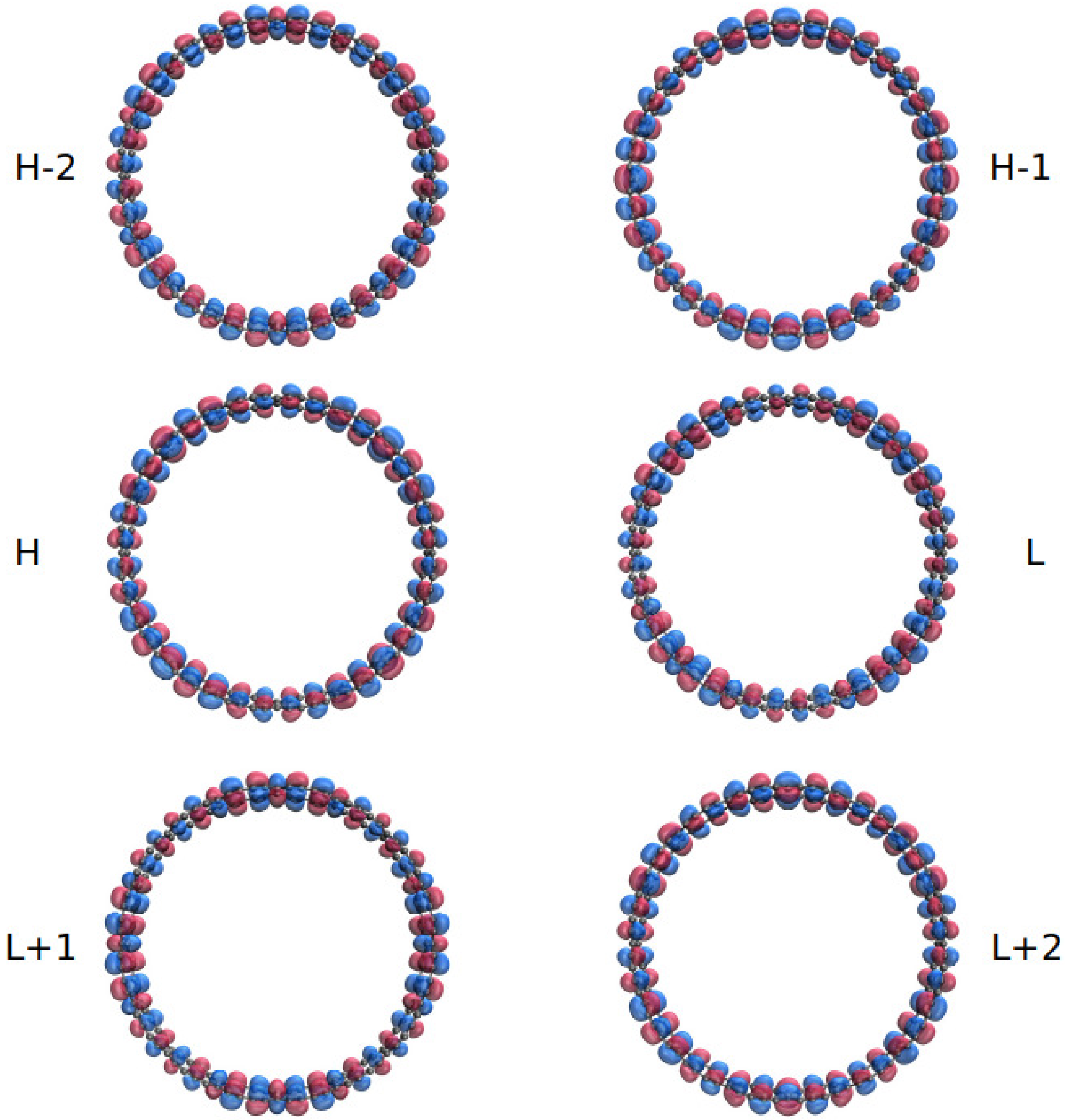

FIG. S3. Visualization of active TAO-orbitals, such as H−2 (1.588), H−1 (1.397), H (1.397), L (0.636), L+1 (0.636), and L+2 (0.373), of ground-state C-Belt[16] at an isovalue of 0.02 e/Å^3, computed using spin-restricted TAO-LDA. Numbers in parentheses show the TOONs.

TABLES

TABLE S1. Singlet-triplet gap E_{ST} (in kcal/mol) of C-Belt[n], computed using spin-unrestricted TAO-LDA.

n	E_{ST}
4	13.88
5	5.06
6	3.71
7	4.07
8	3.52
9	3.34
10	3.23
11	2.74
12	2.48
13	2.31
14	2.14
15	2.01
16	1.90
17	1.78
18	1.68
19	1.59
20	1.51
21	1.44
22	1.38
23	1.32
24	1.26

TABLE S2. Vertical ionization potential IP_v (in eV), vertical electron affinity EA_v (in eV), fundamental gap E_g (in eV), and symmetrized von Neumann entropy S_{vN} of ground-state C-Belt[n], computed using spin-unrestricted TAO-LDA.

n	IP_v	EA_v	E_g	S_{vN}
4	6.80	3.04	3.77	1.64

5	6.38	3.40	2.98	4.75
6	6.31	3.70	2.61	5.83
7	6.23	3.84	2.39	5.84
8	6.13	3.95	2.17	6.64
9	6.06	4.05	2.00	7.28
10	5.99	4.12	1.86	7.78
11	5.92	4.19	1.73	8.85
12	5.87	4.26	1.62	9.73
13	5.83	4.31	1.52	10.48
14	5.79	4.35	1.44	11.30
15	5.76	4.39	1.37	12.06
16	5.73	4.43	1.30	12.84
17	5.70	4.46	1.24	13.66
18	5.67	4.49	1.19	14.46
19	5.65	4.51	1.14	15.26
20	5.63	4.54	1.09	16.06
21	5.61	4.56	1.05	16.86
22	5.59	4.58	1.01	17.66
23	5.57	4.60	0.98	18.46
24	5.56	4.61	0.95	19.26
